# Assessment of the Utilization of Validated Diagnostic Predictive Tools and D-Dimer in the Evaluation of Pulmonary Embolism: A Single-Center Retrospective Cohort Study from a Public Hospital in New York City

**DOI:** 10.3390/jcm12113629

**Published:** 2023-05-23

**Authors:** Amrin Kharawala, Jiyoung Seo, Diego Barzallo, Gabriel Hernandez Romero, Yunus Emre Demirhan, Gustavo J. Duarte, Charan Thej Reddy Vegivinti, Manuel Hache-Marliere, Prasanth Balasubramanian, Heitor Tavares Santos, Sanjana Nagraj, Majd Al Deen Alhuarrat, Dimitrios Karamanis, Dimitrios Varrias, Leonidas Palaiodimos

**Affiliations:** 1 Department of Medicine, New York City Health + Hospitals/Jacobi, Bronx, NY 10461, USA; 2 Albert Einstein College of Medicine, Bronx, NY 10461, USA; 3 Department of Economics, University of Piraeus, 18534 Attica, Greece; 4 Department of Health Informatics, Rutgers School of Health Professions, Newark, NJ 07107, USA; 5 School of Medicine, City University of New York, New York, NY 10031, USA

**Keywords:** pulmonary embolism, computed tomography, CT angiography, D-dimer, Well’s score, revised Geneva score, years algorithm

## Abstract

Introduction: A significant increase in the use of computed tomography with pulmonary angiography (CTPA) for the diagnosis of pulmonary embolism (PE) has been observed in the past twenty years. We aimed to investigate whether the validated diagnostic predictive tools and D-dimers were adequately utilized in a large public hospital in New York City. Methods: We conducted a retrospective review of patients who underwent CTPA for the specific indication of ruling out PE over a period of one year. Two independent reviewers, blinded to each other and to the CTPA and D-dimer results, estimated the clinical probability (CP) of PE using Well’s score, the YEARS algorithm, and the revised Geneva score. Patients were classified based on the presence or absence of PE in the CTPA. Results: A total of 917 patients were included in the analysis (median age: 57 years, female: 59%). The clinical probability of PE was considered low by both independent reviewers in 563 (61.4%), 487 (55%), and 184 (20.1%) patients based on Well’s score, the YEARS algorithm, and the revised Geneva score, respectively. D-dimer testing was conducted in less than half of the patients who were deemed to have low CP for PE by both independent reviewers. Using a D-dimer cut-off of <500 ng/mL or the age-adjusted cut-off in patients with a low CP of PE would have missed only a small number of mainly subsegmental PE. All three tools, when combined with D-dimer < 500 ng/mL or <age-adjusted cut-off, yielded a NPV of > 95%. Conclusion: All three validated diagnostic predictive tools were found to have significant diagnostic value in ruling out PE when combined with a D-dimer cut-off of <500 ng/mL or the age-adjusted cut-off. Excessive use of CTPA was likely secondary to suboptimal use of diagnostic predictive tools.

## 1. Introduction

Pulmonary embolism (PE) is defined as a disruption of blood flow in the pulmonary artery and/or its branches by a thrombus that originated elsewhere in the body [1]. Nearly 900,000 people develop a deep venous thrombosis (DVT) or PE every year in the United States (US) [2]. Due to a large spectrum of non-specific symptoms, PE is considered a great masquerader, which can make definitive diagnosis challenging, with differences in presentation among both males and females [3]. Computed Tomography (CT) angiography of the chest is considered the gold standard diagnostic modality for PE (CT chest with PE protocol/CTPA) [4]. Trends in imaging for suspected PE across US healthcare systems have shown a 450% increase in the use of CTPA with an average annual growth rate of 16.3% per year for years 2004 to 2016 [5]. Due to the increased risk of morbidity and mortality from PE, clinicians tend to err on the side of caution with regard to the diagnosis of PE, which can explain the increase in the use of CTPA [6]. On the other hand, CTPA can lead to adverse effects like contrast-induced nephropathy (CIN) and allergic reactions and increase the risk of radiation-related malignancies [7,8]. The financial burden of ordering CTPA when not indicated could be significant since one CTPA scan costs approximately 651 dollars, while appropriate screening using the D-dimer test ends up costing around 14 dollars [9]. The approximate annual economic burden of CTPA for every 1000 suspected PE patients is approximately 3.8 million dollars [10].

Diagnostic predictive tools based on a combination of clinical features have been developed and validated to assess the probability of PE in adults. Well’s score, the revised Geneva score, and the YEARS algorithm are used to stratify the patients based on the likelihood of having PE [11,12,13,14]. Traditionally, D-dimer has been combined with Well’s score and the YEARS algorithm [11,13,15]. This has been incorporated into the 2019 European Society of Cardiology (ESC) guidelines, where the use of age-adjusted D-dimer is now also encouraged [15]. Despite established algorithms for the diagnosis of PE and the high negative predictive value of D-dimer, studies have found that D-dimer cut-offs are underutilized in the diagnostic process to determine the need for CTPA [6,16,17].

This study aimed to retrospectively evaluate the utilization of validated diagnostic predictive tools and D-dimer in the evaluation for PE in patients that presented at Jacobi Medical Center, one of the largest public hospitals in New York City, USA. We also aimed to compare the sensitivity of validated diagnostic predictive tools along with D-dimer scores to rule out PE in our study population. Through these objectives, we aim to develop an intervention at a later stage with the intention to limit the unnecessary use of CTPA and judiciously use the limited resources in our safety-net public hospital. 

## 2. Materials and Methods

### 2.1. Study Design, Study Setting, and Patient Population

This is a retrospective record-based study on patients who underwent the CTPA at Jacobi Medical Center, one of the largest public hospitals within the New York City Health + Hospitals (NYC H + H). Our system serves a diverse patient population that mainly includes patients with low income (32% uninsured and 35% Medicaid beneficiaries) and/or patients who belong to racial or ethnic minority groups (70%) [18]. All patients ≥ 18 years old who received CTPA using the specific order set to rule out PE between 01 January 2021 and 31 December 2021 in the emergency department and general floors were included. Patients who met any of these criteria were excluded: (i) patients that did not obtain CTPA in the emergency department; (ii) patients < 18 years old; (iii) pregnant women; and (iv) patients undergoing CTPA without the indication of ruling out PE. This study was approved by the Institutional Review Board of the Albert Einstein College of Medicine with a waiver of informed consent (IRB# 2022-13891). The STROBE guidelines for observational studies were followed (Supplementary File S1) [19]. 

### 2.2. Data Sources

The EPIC (Epic systems, Verona, WI, USA) electronic medical record (EMR) was used. Relevant international classification of diseases (ICD) diagnostic codes and search terms were used, and anonymized data were extracted. 

Data were extracted by retrospective review of patient charts by four independent researchers who were blinded to each other (AK, JS, YD, GD; group A). Another researcher (MA) cross-checked the data. Discrepancies were resolved by reaching consensus. Study definitions for variables extracted are provided in Supplementary File S2. The data on baseline patient characteristics including age, sex, race, body mass index (BMI), symptoms including dyspnea, chest pain, cough, hemoptysis, palpitations, lightheadedness/presyncope/syncope, basic vital signs including temperature, heart rate, respiratory rate, blood pressure, and oxygen saturation (triage vital signs), and comorbidities including diabetes mellitus, hypertension, heart failure, chronic kidney disease, anemia, chronic obstructive pulmonary disease (COPD), and asthma were collected. Further data on lower extremity venous duplex findings, chest X-ray, electrocardiogram (EKG), transthoracic echocardiography (TTE), troponin, pro-Brain Natriuretic Peptide (pro-BNP) levels within 72 h before or after CTPA, as well as development of post-CTPA acute kidney injury (AKI), were collected. 

Another team of reviewers (CV-DB-GH (group B1) and PB-MH-HT (group B2)) blinded to each other was responsible for assessing the clinical probability (CP) of PE retrospectively. All group B reviewers were provided envelopes with all available documentation, clinical, laboratory, and radiologic data that preceded the order time of CTPA (data from triage time to the CTPA order time for patients that received CTPA) but no information on the results of CTPA, venous duplex, D-dimer testing, and any documentation indicating the results of these tests. Every patient envelope was reviewed by two independent blinded reviewers (one from group B1 and another from group B2) who calculated the scores using the three validated predictive tools (Well’s score, YEARS algorithm, revised Geneva score) to assess the CP of PE. Patients were considered to have low CP only if the scores assigned by both independent blinded reviewers (one from each groups: B1 and B2) fell under the ‘low risk category’ for Well’s score and revised Geneva score and for YEARS score of zero (Supplementary File S2). 

### 2.3. Exposure of Interest and Outcomes

The primary outcome was CTPA obtained in patients who were deemed to be low risk for PE with or without D-dimer testing. The secondary outcomes were (i) diagnostic accuracy (sensitivity, specificity, negative predictive value, and positive predictive value) of validated diagnostic predictive tools in our patient population and (ii) diagnostic accuracy of combining different D-dimer cut-offs with Well’s score, YEARS algorithm, and revised Geneva score to safely rule out PE.

### 2.4. Statistical Analysis

Patients were classified into two groups based on the results of CTPA: PE present and PE absent. The absolute and relative rates of patients with “PE unlikely” or “PE likely” (two tiers) per Well’s score and revised Geneva score and the mean and median numbers of present criteria per YEARS algorithm were calculated. For patients who received D-dimer tests, the absolute and relative rates of patients with D-dimer levels of <500 ng/mL, <1000 ng/mL, and <age-adjusted D-dimer cut-off (age × 10 cut-off for age >50 years) were calculated for all subgroups [12]. Subgroup analysis was performed in respect to COVID-19 status. The sensitivity, specificity, negative predictive value, and positive predictive value for the diagnosis of PE were calculated for all subgroups. The area under the curve (AUC) of the receiver operating characteristics (ROC) was calculated in order to measure the effectiveness of diagnostic tools. 

Continuous data were presented as median with IQR and categorical data as absolute and relative frequencies. The ANOVA test was used to compare the continuous variables, while chi-square was used for discrete variables. Interaction analyses were performed as needed. The threshold of statistical significance was set at *p* ≤ 0.05. All analyses were performed using STATA software (version 14·1; STATA Corporation, College Station, TX, USA).

## 3. Results

### 3.1. Patient Characteristics, Symptoms, Signs, Cardiac Markers, and Imaging Findings

A total of 917 patients (female: 541, median age: 57 years) fulfilled the study criteria, among whom 128 patients were found to have PE. The median age of patients who had PE was significantly higher than those without PE (61 vs. 56 years; *p* < 0.001). Apart from a higher prevalence of a past medical history of DVT/PE in patients with PE (26.6% vs. 15%; *p* = 0.001), no other significant association was noted with regard to medical history (Table 1). The presence of pain or edema on a unilateral limb was significantly more frequent in patients with PE (25% vs. 12.8%; *p* < 0.001). On the other hand, chest pain was more common in the non-PE group (44.7% vs. 28.9%; *p* = 0.001). No other significant associations were noted in respect to symptoms (Table 2). Regarding vital signs, a higher respiratory rate (19 vs. 18 per minute; *p* < 0.001) and a lower oxygen saturation (96% vs. 97% *p* = 0.002) were noted in the PE group (Table 2). Troponin elevation (14.7 vs. 5.9%; *p* = 0.001) and pro-BNP elevation (59.8% vs. 48.9%; *p* = 0.046) were significantly more frequent in the PE group. 46/128 (35.9%) patients with PE were found to have a DVT on the lower extremity venous duplex, as opposed to 26/789 (32.9%) patients without PE (*p* < 0.001). Right heart dysfunction on TTE was significantly more common in patients with PE (25.58% vs. 7.81%; *p* < 0.001) (Table 3). 

### 3.2. Retrospective Assessment of Clinical Probability Based on Diagnostic Predictive Tools

The CP of PE was considered low by both independent reviewers in 563 (61.4%), 487 (55%), and 184 (20.1%) patients based on Well’s score, the YEARS algorithm, and the revised Geneva score, respectively. D-dimer testing was conducted in less than half of patients who were deemed to have a low CP of PE by both independent reviewers (Well’s: 40%), YEARS: 39.6%, and Geneva: 48.4%).

Based on Well’s score, of 122/225 (54.2%) low-risk patients with a D-dimer <500 ng/mL, 5 patients (4.1%) were diagnosed with PE (3 sub-segmental PE; 2 segmental PE). As per the YEARS algorithm, of 102/193 (52.9%) low CP patients with a D-dimer <500 ng/mL, 4 patients (3.9%) were diagnosed with PE (3 sub-segmental PE; 1 segmental PE). Based on the revised Geneva score, of 55/89 (61.8%) low CP patients with a D-dimer of <500 ng/mL, 3 patients (5.5%) were diagnosed with PE (all three with sub-segmental PE). None of the PE in these patients was massive or larger than segmental. Similar findings were observed when the age-adjusted D-dimer cut-off was applied.

The retrospective assessment of CP based on diagnostic predictive tools with and without various D-dimer cut-offs is presented in Table 4. A sensitivity analysis that included only patients without COVID-19 (792/917 patients, 84.6% of the total cohort) yielded similar findings (Supplementary Material S3). 

Patients that were found to have PE had a mean Well’s score of 2.88, while patients without PE had a mean score of 2.18 (*p* < 0.001). Regarding the YEARS algorithm, the mean number of positive items was 0.49 in the PE group versus 0.32 in the no-PE group (*p* = 0.002). 

Patients that were found to have PE had a mean revised Geneva score of 6.99, while patients without PE had a mean score of 5.68 (*p* < 0.001).

The median D-dimer value was 490 ng/mL. Patients that were found to have PE had a median D-dimer value of 1956 ng/mL versus 418 ng/mL in the patients without PE (*p* < 0.001). Only 5.1% (9/175) of patients with D-dimer <500 ng/mL were found to have PE (subsegmental: 6, segmental: 3, and other: 0). Similarly, 5% (10/201) of patients with D-dimer < age-adjusted cut-off were found to have PE (subsegmental: 6, segmental: 3, and other: 1). Detailed data on D-dimer are presented in Supplementary File S4. The characteristics of the patients who had PE despite low clinical probability and negative D-dimer are described in Supplementary File S5.

The combination of D-dimer < 500 ng/mL and classification as low CP for PE by both independent reviewers based on Well’s score, YEARS algorithm, and modified Geneva score were estimated to yield sensitivity to rule out PE of 82%, 83%, and 75%, respectively. The results were found to be the same when the D-dimer cut-off was the age-adjusted one. All the above combinations yielded NPV > 95%. Specificity, sensitivity, PPV, and NPV for all combinations of diagnostic tools are presented in Table 5. 

## 4. Discussion

Our study evaluated the utilization and the accuracy of common diagnostic predictive tools along with D-dimer testing for the diagnosis of pulmonary embolism in a cohort of 917 patients in a public hospital in New York City. It was observed that a significant proportion of patients that underwent CTPA were deemed to be of a low CP of PE retrospectively, and D-dimer testing was conducted in less than half of the patients with a low CP of PE. Using a D-dimer less than the age-adjusted cut-off in low CP patients would have missed only a small number of mainly subsegmental PE. Well’s score, the YEARS algorithm, and the modified Geneva score, when combined with D-dimer, yielded NPV > 95%. 

Well’s score, the YEARS algorithm, and the modified Geneva score are commonly used validated diagnostic predictive tools with decent sensitivity to rule out PE, especially when combined with low D-dimer [11,12,13,20,21]. The findings of our study indicated that these tools were underutilized during the study period, potentially leading to excessive chest imaging. A significant percentage of patients who were considered to be of a low CP of PE by both independent reviewers using Well’s score, the YEARS algorithm, and the revised Geneva score were subjected to CTPA (61.4%, 55%, and 20.1%, respectively), and D-dimer testing was conducted in less than half of low-CP patients. Underutilization of the validated diagnostic predictive tools and D-dimer and overuse of CTPA are common in clinical practice [22,23,24]. Retrospective studies have reported the use of a clinical predictive tool in only 15–60% of patients with suspected PE [22,23,25,26]. Similarly, the underutilization of D-dimer assay has been a challenge in many hospital settings across the United States and in Europe [23,27,28,29,30]. The overuse of CTPA has been estimated at 18.7 to 88% in resourceful settings [24,30,31,32]. Underfunded institutions that primarily serve underinsured patients, such as our public hospital, need to use their resources wisely [33]. This study took steps to identify the extent of the problem and can be the foundation for the implementation of interventions designed to increase the awareness of physicians towards diagnostic predictive tools and D-dimer use, which can lead to a safe reduction in chest imaging. 

Our study findings confirmed that all three predictive tools have excellent and comparable negative predictive value when combined with D-dimer regardless of the D-dimer threshold [14,34]. The highest sensitivity to rule out PE was observed with the combination of low CP per YEARS algorithm with D-dimer < age-adjusted cut-off (83%) and the combination of low CP per Well’s score and D-dimer < age-adjusted cut-off (82%). The combination of low CP per modified Geneva score and D-dimer < age-adjusted cut-off was found to yield lower sensitivity (75%), which is consistent with the literature [35]. It is important to be noted that all three tools were estimated to have a sensitivity of < 50% to rule out PE when used without D-dimer in our study; hence, they should not be used to rule out PE without being combined with low D-dimer [36].

It was very reassuring that only a small fraction of patients (3.3–5.3% depending on the predictive tool) found to have a low CP of PE and D-dimer < age-adjusted cut-off were diagnosed with PE based on CTPA. Most of these patients were found to have subsegmental PE. Therefore, only a few cases, of mainly subsegmental PE, would have been missed should the tools have been applied in conjunction with D-dimer testing. It is important to mention that subsegmental PE is frequently over-diagnosed [37,38,39]; thus, it is likely that some of the few subsegmental PE missed were not actual PE but false positive findings. On expert review, nearly 30% of subsegmental PEs were deemed to be indeterminate, while up to 15% were considered to be false positives for PE, mainly due to respiratory motion artifacts [40,41]. Moreover, it is not clear whether the benefits of anticoagulation outweigh the risks in patients with subsegmental PE, particularly in the absence of cancer, pregnancy, or deep vein thrombosis [41,42].

We acknowledge that our study has some limitations. First, this was a retrospective cohort involving medical records; hence, there are risks related to observational bias and unmeasured confounding. It is challenging to assess the clinical probability of PE retrospectively. Therefore, we employed a very robust methodology of independent blinded reviews, and we considered a low CP of PE only in those cases where both reviewers were in agreement. Second, this was a single-center study conducted in a public hospital in NYC; therefore, our findings cannot be easily generalized to other settings. On the other hand, our study has several strengths. First, we assessed the underutilization of clinical tools for the assessment of CP of PE and, thus, the overuse of chest imaging in a setting with limited resources. Second, a thorough chart review was conducted involving teams of independent reviewers blinded to key results and to each other. Third, we examined all three common diagnostic tools.

In conclusion, a significant proportion of patients underwent CTPA despite having a low CP of PE due to suboptimal utilization of validated diagnostic predictive tools and D-dimer testing, potentially leading to excessive chest imaging. It was found that the use of Well’s score, the YEARS algorithm, or the modified Geneva score in combination with the D-dimer < age-adjusted cut-off offers decent sensitivity to rule out PE and an excellent NPV. Clinicians should be encouraged to use diagnostic predictive tools to assess the CP of PE in all patients in the ER and on general floors when PE is in the differential diagnosis, along with D-dimer testing when CP is deemed to be low. Interventions aiming to improve the utilization of validated diagnostic predictive tools and D-dimer and decrease unnecessary chest imaging should be designed and implemented, particularly in settings with limited resources. 

## Figures and Tables

**Table 1:** Baseline Patient Characteristics.

		Pulmonary Embolism		
	Total	No	Yes	
	N = 917	N = 789	N = 128	*p*-Value
Gender				0.099
Male	376 (41)	315 (39.9)	61 (47.7)	
Female	541 (59)	474 (60.1)	67 (52.3)	
Age	57 (41–68)	56 (39–68)	61 (47–73)	<0.001
Body Mass Index	29.1 (24.2–35.4)	29 (24.2–35.3)	29.9 (23.7–35.5)	0.659
Race/Ethnicity				0.159
African American	375 (41)	319 (40.5)	56 (43.7)	
Hispanic/Latinos	308 (33.7)	259 (32.9)	49 (38.2)	
White	108 (11.8)	95 (12.1)	13 (10.2)	
Other	124 (13.5)	114 (14.5)	10 (7.8)	
Diabetes Mellitus				0.274
No	688 (75)	587 (74.4)	101 (78.9)	
Yes	229 (25)	202 (25.6)	27 (21.1)	
Hypertension				0.180
No	473 (51.6)	414 (52.5)	59 (46.1)	
Yes	444 (48.4)	375 (47.5)	69 (53.9)	
Heart Failure				0.087
No	829 (90.4)	708 (89.7)	121 (94.5)	
Yes	88 (9.6)	81 (10.3)	7 (5.5)	
COPD or Asthma				0.814
No	693 (76.2)	596 (76)	97 (77)	
Yes	217 (23.8)	188 (24)	29 (23)	
CKD or ESRD				0.406
No	867 (94.6)	744 (94.3)	123 (96.1)	
Yes	50 (5.4)	45 (5.7)	5 (3.9)	
Hemoglobin	12.5 (10.6–13.8)	12.5 (10.6–13.8)	12.5 (10.6–13.8)	0.812
SARS-CoV-2 infection				0.667
No	792 (86.4)	683 (86.6)	109 (85.2)	
Yes	125 (13.6)	106 (13.4)	19 (14.8)	
History of VTE				0.001
No	765 (83.4)	671 (85)	94 (73.4)	
Yes	152 (16.6)	118 (15)	34 (26.6)	
Active malignancy				0.158
No	819 (89.4)	700 (88.8)	119 (93)	
Yes	97 (10.6)	88 (11.2)	9 (7)	

Notes: All variables are expressed in n (%) except for age, body mass index, and hemoglobin, which are expressed in median (IQR). Abbreviations: CKD: chronic kidney disease; COPD: chronic obstructive lung disease; ESRD: end-stage renal disease; IQR: interquartile range; N: number; SARS-CoV2: severe acute respiratory syndrome-coronavirus 2; VTE: venous thromboembolism; and %: percentage.

**Table 2:** Symptoms and signs.

		Pulmonary Embolism		
	Total	No	Yes	
	N = 917	N = 789	N = 128	*p*-Value
Chest pain				0.001
No	527 (57.5)	436 (55.3)	91 (71.1)	
Yes	390 (42.5)	353 (44.7)	37 (28.9)	
Dyspnea				0.366
No	382 (41.7)	324 (41.1)	58 (45.3)	
Yes	535 (58.3)	465 (58.9)	70 (54.7)	
Cough				0.047
No	664 (72.4)	562 (71.2)	102 (79.7)	
Yes	253 (27.6)	227 (28.8)	26 (20.3)	
Palpitations				0.558
No	747 (81.6)	645 (81.8)	102 (79.7)	
Yes	169 (18.4)	143 (18.2)	26 (20.3)	
Dizziness or syncope				0.604
No	788 (86)	676 (85.8)	112 (87.5)	
Yes	128 (14)	112 (14.2)	16 (12.5)	
Hemoptysis				0.409
No	882 (96.4)	757 (96.1)	125 (97.7)	
Yes	33 (3.6)	30 (3.8)	3 (2.3)	
Fever				0.517
No	792 (86.5)	679 (86.2)	113 (88.3)	
Yes	124 (13.5)	109 (13.8)	15 (11.7)	
LE pain or				<0.001
U/L edema	788 (86)	692 (87.8)	96 (75)	
No	128 (14)	96 (12.2)	32 (25)	
Yes	100 (85–115)	100 (84–114)	101 (86–118)	0.071
HR	18 (18–20)	18 (18–20)	19 (18–20)	<0.001
RR	98.3 (98–98.8)	98.3 (98–98.8)	98.4 (98–99)	0.288
Temperature	131 (117–146)	131 (117–146)	131 (112.5–149)	0.393
SBP	79 (72–88)	79 (72–87)	80.5 (70.5–89.5)	0.783
DBP	97 (94–99)	97 (95–99)	96 (92–98)	0.002

Notes: All variables are expressed in n (%) except for HR, RR, temperature, SBP, DBP, and SO2, which are expressed in median (IQR). Abbreviations: DBP: diastolic blood pressure; HR: heart rate; IQR: interquartile range; LE: lower extremity; N: number; RR: respiratory rate; SBP: systolic blood pressure; SO2: oxygen saturation, U/L: unilateral; and %: percentage.

**Table 3:** Cardiovascular work-up.

		Pulmonary Embolism		
	Total	No	Yes	
	N = 917	N = 789	N = 128	*p*-Value
EKG changes				0.343
NSR	422 (51.1)	365 (51.8)	57 (47.1)	
Sinus tachycardia or afib/a-flutter	404 (48.9)	340 (48.2)	64 (52.9)	
LE venous duplex for DVT				<0.001
No DVT	185 (72)	141 (84.4)	44 (48.9)	
Unilateral DVT	51 (19.8)	18 (10.8)	33 (36.7)	
Bilateral DVT	21 (8.2)	8 (4.8)	13 (14.4)	
TTE				<0.001
No acute RV dysfunction *	264 (87.1)	200 (92.2)	64 (74.4)	
Acute RV dysfunction present	39 (12.9)	17 (7.8)	22 (25.6)	
Elevated Troponin				0.001
No	698 (92.7)	599 (94)	99 (85.3)	
Yes	55 (7.3)	38 (6)	17 (14.7)	
Elevated pro-BNP				0.046
No	265 (49)	224 (51.1)	41 (40.2)	
Yes	275 (51)	214 (48.9)	61 (59.8)	

Notes: All variables are expressed in n (%). Abbreviations: A-flutter: atrial flutter; Afib: atrial fibrillation; DVT: deep venous thrombosis; EKG: electrocardiogram; LE: lower extremity; NSR: normal sinus rhythm; pro-BNP: pro-brain natriuretic peptide; RV: right ventricle; TTE: transthoracic echocardiogram; and %: percentage. Troponin (Normal: 0.000–0.090 ug/L); ProBNP (Normal: 1–450 pg/mL); and * RV dysfunction is based on acute RV pressure overload in the setting of acute PE (used to classify it as a sub-massive PE).

**Table 4:** (**a**) Retrospective assessment of validated diagnostic predictive tools; (**b**) retrospective assessment of validated diagnostic predictive tools with D-dimer.

(a)									
		Pulmonary Embolism			Pulmonary Embolism Present				
	Total	No	Yes		Total	Subsegmental PE	Segmental PE	Other PE	
	N = 917	N = 789	N = 128	*p*-Value	N = 128	N = 36	N = 56	N = 36	*p*-Value
Well’s score: reviewer 1/reviewer 2				<0.001					0.356
Unlikely/unlikely	563 (61.4)	506 [89.9]	57 [10.1]		57 (46.30)	16 [28.1]	28 [49.1]	13 [22.8]	
Likely/unlikely or vice versa	212 (24)	178 [83.9]	34 [16]		34 (27.6)	9 [26.5]	11 [32.4]	14 [41.1]	
Likely/likely	109 (12.3)	77 [70.6]	32 [29.4]		32 (26)	8 [25]	16 [50]	8 [25]	
YEARS algorithm: reviewer 1/reviewer 2				<0.001					0.902
0 items/0 items	487 (55)	440 [90.4]	47 [9.7]		47 (38.2)	14 [29.8]	21 [44.7]	12 [25.5]	
≥1 items/0 items or vice versa	245 (27.7)	207 [84.5]	38 [15.5]		38 (30.9)	10 [26.3]	15 [39.5]	13 [34.2]	
≥items/≥ items	153 (17.3)	115 [75.2]	38 [24.8]		38(30.9)	10 [26.3]	18 [47.4]	10 [26.3]	
Revised Geneva score (same for both reviewers)				0.001					0.928
Low	184 (20.1)	166 [90.2]	18 [9.8]		18 (14.1)	5 [27.8]	8 [44.4]	5 [27.8]	
Intermediate	662 (72.4)	571 [86.3]	91 [13.8]		91 (71.1)	26 [28.6]	38 [41.8]	27 [29.6]	
High	69 (7.5)	50 [72.5]	19 [27.5]		19 (14.8)	5 [26.3]	10 [52.6]	4 [21.1]	
**(b)**									
Well’s score: unlikely/unlikely	N = 563				N = 57				0.270
D-dimer cut-off: 1000				<0.001					
<1000	168 (74.7)	158 [94.1]	10 [5.9]		10 (37)	5 [50]	3 [30]	2 [20]	
≥1000	57 (25.3)	40 [70.2]	17 [29.9]		17 (63)	3 [17.7]	9 [52.9]	5 [29.4]	0.222
D-dimer cut-off: 500				<0.001					
<500	122 (54.2)	117 [95.9]	5 [4.1]		5 (18.5)	3 [60]	2 [40]	0 [0]	
>500	103 (45.8)	81 [78.6]	22 [21.4]		22 (81.5)	5 [22.7]	10 [45.5]	7 [31.8]	0.222
D-dimer cut-off: age-adjusted				<0.001					
<age-adjusted	142 (63.1)	137 [96.5]	5 [3.5]		5 (18.5)	3 [60]	2 [40]	0 [0]	
>age-adjusted	83 (36.9)	61 [73.5]	22 [26.5]		22 (81.5)	5 [22.7]	10 [45.5]	7 [31.8]	
YEARS algorithm: 0 items/0 items	N = 487				Ν = 47				0.275
D-dimer cut-off: 1000				<0.001					
<1000	144 (74.6)	135 [93.8]	9 [6.2]		9 (39.1)	5 [55.6]	2 [22.2]	2 [22.2]	
≥1000	49 (25.4)	35 [71.4]	14 [28.6]		14 (60.9)	3 (21.4)	7 [50]	4 [28.6]	0.261
D-dimer cut-off: 500				<0.001					
<500	102 (52.9)	98 [96.1]	4 [3.9]		4 (17.4)	3 [75]	1 [25]	0 [0]	
>500	91 (47.2)	72 [79.1]	19 [20.9]		19 (82.6)	5 [26.3]	8 [42.1]	6 [31.6]	0.261
D-dimer cut-off: age-adjusted				<0.001					
<age-adjusted	121 (62.7)	117 [96.7]	4 [3.3]		4 (17.4)	3 [75]	1 [25]	0 [0]	
>age-adjusted	72 (37.3)	53 [73.6]	19 [26.4]		19 (82.6)	5 [26.3]	8 [42.1]	6 [31.6]	
Revised Geneva score: low risk	N = 184				Ν = 18				0.356
D-dimer cut-off: 1000				0.008					
<1000	67 (75.3)	62 [92.5]	5 [7.5]		5 (41.7)	3 [60]	1 [20]	1 [20]	
≥1000	22 (24.7)	15 [68.2]	7 [31.8]		7 (58.3)	1 [14.3]	4 [57.1]	2 [28.6]	0.023
D-dimer cut-off: 500				0.009					
<500	55(61.8)	52 [94.6]	3 [5.5]		3 (25)	3 [100]	0 [0]	0 [0]	
>500	34 (38.2)	25 [73.5]	9 [26.5]		9 (75)	1 [11.1]	5 [55.6]	3 [33.3]	0.023
D-dimer cut-off: age-adjusted				0.007					
<age-adjusted	57 (64)	54 [94.7]	3 [5.3]		3 (25)	3 [100]	0 [0]	0 [0]	
>age-adjusted	32 (36)	23 [71.9]	9 [28.1]		9 (75)	1 [11.1]	5 [55.6]	3 [33.3]	

(**a**) Notes: (1) All variables are expressed in n (%) or [%]; (%) corresponds to columns and [%] to rows; (2) Well’s score ≤ 4: PE unlikely; (3) revised Geneva Score 0–3: low risk for PE; and (4) for Well’s criteria and YEARS algorithm, clinical probability for PE as independently assessed by reviewer 1 and reviewer 2 are presented as ‘reviewer 1/reviewer 2′. Abbreviations: N: number and PE: pulmonary embolism. (**b**) Notes: (1) All variables are expressed in n (%) or [%]; (%) corresponds to columns and [%] to rows; (2) D-dimer is in ng/mL; (3) Well’s score ≤ 4: PE unlikely; (4) revised Geneva Score 0–3: low risk for PE; (5) for Well’s criteria and YEARS algorithm, clinical probability for PE as independently assessed by reviewer 1 and reviewer 2 are presented as ‘reviewer 1/reviewer 2′; and (6) Age-adjusted D-dimer (Age × 10 microgram/Liter if age > 50 years). Abbreviations: N: number and PE: pulmonary embolism.

**Table 5:** Diagnostic accuracy of predictive tools.

Predictors of PE	Sensitivity	Specificity	PPV	NPV	AUC	Observations
Well’s -/-	46%	34%	10%	80%	0.40	884
YEARS -/-	38%	42%	10%	81%	0.40	885
Geneva low	14%	79%	10%	85%	0.47	915
Well’s -/- and D-dimer < 500	82%	59%	21%	96%	0.70	225
Well’s -/- and D-dimer < age-adjusted D-dimer	82%	69%	27%	97%	0.75	225
Well’s -/- and D-dimer < 1000	61%	79%	29%	94%	0.71	225
YEARS -/- and D-dimer < 500	83%	58%	21%	96%	0.71	193
YEARS -/- and D-dimer < age-adjusted D-dimer	83%	69%	26%	97%	0.76	193
YEARS -/- and D-dimer < 1000	61%	79%	29%	94%	0.71	193
Geneva low and D-dimer < 500	75%	68%	27%	95%	0.71	89
Geneva low and D-dimer < age-adjusted D-dimer	75%	70%	28%	95%	0.73	89
Geneva low and D-dimer < 1000	58%	81%	32%	93%	0.69	89

**Notes:** (1) D-dimer is in ng/mL; (2) Well’s score ≤ 4: PE unlikely; (3) revised Geneva Score 0–3: low risk for PE; and (4) Age-adjusted D-dimer (Age × 10 microgram/Liter if age > 50 years). Abbreviations: PE: pulmonary embolism, PPV: Positive predictive value, NPV: Negative predictive value, and AUC: area under the curve.

## Data Availability

The data that support the findings of this study are available in the Supplementary Material of this article.

## Supplementary Materials

The following supporting information can be downloaded at https://www.mdpi.com/article/10.3390/jcm12113629/s1. Supplementary File S1: STROBE check list, Supplementary File S2: Study Definitions; Supplementary File S3: Sensitivity analysis for Non-COVID patients; Supplementary File S4: D-dimer and PE; Supplementary File S5: Patients with low clinical probability and D-dimer <500.
