# Supplementary material for: Assessment of the Utilization of Validated Diagnostic Predictive Tools and D-Dimer in the Evaluation of Pulmonary Embolism: A Single-Center Retrospective Cohort Study from a Public Hospital in New York City
Source: J Clin Med. 2023 May 23;12(11):3629. doi: 10.3390/jcm12113629 (PMC10253672; doi:10.3390/jcm12113629)
Supplement: Supplementary file 1 [file jcm-12-03629-s001.zip › jcm-2352864-supplementary.pdf]

Supplementary File S1

STROBE Statement—Checklist of items that should be included in reports of ***cohort studies.***

	Item No	Recommendation	Page No.
**Title/abstract**	1	(*a*) Indicate the study's design with a commonly used term in the title or the abstract	1
		(*b*) Provide in the abstract an informative and balanced summary of what was done and what was found	4,5
**Introduction**			
Background/rationale	2	Explain the scientific background and rationale for the investigation being reported	6,7
Objectives	3	State specific objectives, including any prespecified hypotheses	9
**Methods**			
Study design	4	Present key elements of study design early in the paper	7-9
Setting	5	Describe the setting, locations, and relevant dates, including periods of recruitment, exposure, follow-up, and data collection	7-9
Participants	6	(*a*) Give the eligibility criteria, and the sources and methods of selection of participants. Describe methods of follow-up	7-9
		(*b*) For matched studies, give matching criteria and number of exposed and unexposed	
Variables	7	Clearly define all outcomes, exposures, predictors, potential confounders, and effect modifiers. Give diagnostic criteria, if applicable	7-9, Supplementary material 2
Data sources/ measurement	8*	For each variable of interest, give sources of data and details of methods of assessment (measurement). Describe comparability of assessment methods if there is more than one group	
Bias	9	Describe any efforts to address potential sources of bias	7-9
Study size	10	Explain how the study size was arrived at	7-9
Quantitative variables	11	Explain how quantitative variables were handled in the analyses. If applicable, describe which groupings were chosen and why	7-9
Statistical methods	12	(*a*) Describe all statistical methods, including those used to control for confounding	9
		(*b*) Describe any methods used to examine subgroups and interactions	9
		(*c*) Explain how missing data were addressed	9

		(*d*) If applicable, explain how loss to follow-up was addressed	9
		(*e*) Describe any sensitivity analyses	9, Supplementary material 3
**Results**			
Participants	13*	(a) Report numbers of individuals at each stage of study—eg numbers potentially eligible, examined for eligibility, confirmed eligible, included in the study, completing follow-up, and analysed	10
		(b) Give reasons for non-participation at each stage	N/A
		(c) Consider use of a flow diagram	N/A
Descriptive data	14*	(a) Give characteristics of study participants (eg demographic, clinical, social) and information on exposures and potential confounders	Table 1, 10
		(b) Indicate number of participants with missing data for each variable of interest	Table 1
		(c) Summarise follow-up time (eg, average and total amount)	N/A
Outcome data	15*	Report numbers of outcome events or summary measures over time	11,12
Main results	16	(*a*) Give unadjusted estimates and, if applicable, confounder-adjusted estimates and their precision (eg, 95% confidence interval). Make clear which confounders were adjusted for and why they were included	11,12
		(*b*) Report category boundaries when continuous variables were categorized	11,12
		(*c*) If relevant, consider translating estimates of relative risk into absolute risk for a meaningful time period	N/A
Other analyses	17	Report other analyses done—eg analyses of subgroups and interactions, and sensitivity analyses	Supplementary material 3
**Discussion**			
Key results	18	Summarise key results with reference to study objectives	12
Limitations	19	Discuss limitations of the study, taking into account sources of potential bias or imprecision. Discuss both direction and magnitude of any potential bias	14
Interpretation	20	Give a cautious overall interpretation of results considering objectives, limitations, multiplicity of analyses, results from similar studies, and other relevant evidence	12-15
Generalisability	21	Discuss the generalisability (external validity) of the study results	14,15
**Other information**			

Funding	22	Give the source of funding and the role of the funders for the present study and, if applicable, for the original study on which the present article is based	1

*Give information separately for exposed and unexposed groups.

Note: An Explanation and Elaboration article discusses each checklist item and gives methodological background and published examples of transparent reporting. The STROBE checklist is best used in conjunction with this article (freely available on the Web sites of PLoS Medicine at http://www.plosmedicine.org/, Annals of Internal Medicine at http://www.annals.org/, and Epidemiology at http://www.epidem.com/). Information on the STROBE Initiative is available at http://www.strobe-statement.org.

Supplementary File S2

Study definitions

1. **Wells score:** Devised in 2000 using logistic regression analysis, the Well's score uses seven variables (points): clinic symptoms of DVT (3 points), no alternative diagnosis (3 points), heart rate >100 (1.5 points), immobilization or surgery in the previous 4 weeks (1.5 points), previous DVT/PE (1.5 points), hemoptysis (1 point) and malignancy (1 point), to classify patients as low risk (score <2), moderate risk (score of 2–6) and high risk (score >6)[1,2].
2. **The revised Geneva score:** Devised in 2006 using logistic regression analysis, the revised Geneva score uses eight variables (points): age older than 65 years (1 point), previous deep venous thrombosis or PE (3 points), surgery or fracture within 1 month (2 points), active malignant condition (2 points), unilateral lower limb pain (3 points), hemoptysis (2 points), heart rate of 75–94 beats/min (3 points) or 95 beats/min or more (5 points), and pain on lower-limb deep venous palpation and unilateral edema (4 points), to classify patients as low risk (0–3 points), intermediate risk (4–10 points), and high risk (score ≥11 points)[3].
3. **The YEARS algorithm**: Devised in 2017, the YEARS algorithm uses three variables with one point each: clinical signs of deep vein thrombosis, hemoptysis, and if pulmonary embolism is the most likely diagnosis to stratify into YEARS with a score of zero vs YEARS with a score of >=1[4].
4. **Diagnostic Predictive Tools**: Includes the Well's score, revised Geneva score, and YEARS algorithm. These tools help to risk stratify patients with suspected PE into different risk categories, which can then help guide diagnostic algorithms.

Search Definition

The combination of keywords that constituted our search algorithm was Base: "All Patients," Location: "Jacobi Medical Center," and any of the following: 'Original Procedure: CT Chest Pulmonary Embolism with contrast' or 'Procedure: CT Chest Pulmonary Embolism with contrast' or 'Original Procedure: CT Angio Pulmonary Embolism with contrast' or 'Procedure: CT Angio Pulmonary Embolism with contrast'.

References

1. Wells PS, Anderson DR, Rodger M, Ginsberg JS, Kearon C, Gent M, Turpie AG, Bormanis J, Weitz J, Chamberlain M, et al. Derivation of a simple clinical model to categorize patients probability of pulmonary embolism: increasing the models utility with the SimpliRED D-dimer. *Thromb Haemost*. 2000;83:416-420.
2. Wells PS, Anderson DR, Rodger M, Stiell I, Dreyer JF, Barnes D, Forgie M, Kovacs G, Ward J, Kovacs MJ. Excluding pulmonary embolism at the bedside without diagnostic imaging: management of patients with suspected pulmonary embolism presenting to the emergency department by using a simple clinical model and d-dimer. *Ann Intern Med*. 2001;135:98-107. doi: 10.7326/0003-4819-135-2-200107170-00010
3. Le Gal G, Righini M, Roy PM, Sanchez O, Aujesky D, Bounameaux H, Perrier A. Prediction of pulmonary embolism in the emergency department: the revised Geneva score. *Ann Intern Med*. 2006;144:165-171. doi: 10.7326/0003-4819-144-3-200602070-00004
4. van der Hulle T, Cheung WY, Kooij S, Beenen LFM, van Bemmel T, van Es J, Faber LM, Hazelaar GM, Heringhaus C, Hofstee H, et al. Simplified diagnostic management of suspected pulmonary embolism (the YEARS study): a prospective, multicentre, cohort study. *Lancet*. 2017;390:289-297. doi: 10.1016/S0140-6736(17)30885-1

Supplementary File S3: Sensitivity Analysis for Non-COVID patients									
	Pulmonary Embolism				Pulmonary Embolism Present				
	Total	No	Yes		Total	Subsegmental PE	Segmental PE	Other PE	
	N=792	N=683	N=109	p-value	N=109	N=32	N=46	N=31	p-value
Well's score: reviewer 1 / reviewer 2				<0.001					0.358
Unlikely/unlikely	480 (62.8)	436 [90.8]	44 [9.2]		44 (42.3)	13 [29.6]	22 [50]	9 [20.4]	
Likely/unlikely or vice versa	190 (24.8)	159 [83.7]	31 [16.3]		31 (29.8)	8 [25.8]	10 [32.3]	13 [41.9]	
Likely/likely	95 (12.4)	66 [69.4]	29 [30.5]		29 (27.9)	8 [27.6]	13 [44.8]	8 [27.6]	
YEARS algorithm: reviewer 1 / reviewer 2				<0.001					0.923
0 items / 0 items	419 (54.7)	382 [91.2]	37 [8.8]		37 (35.6)	11 [29.7]	17 [46]	9 [24.3]	
≥ 1 items / 0 items or vice versa	218(28.5)	186 [85.3]	32 [14.7]		32 (30.8)	9 [28.1]	12 [37.5]	11 [34.4]	
≥ items / ≥ items	129 (16.8)	94 [72.9]	35 [27.1]		35 (33.7)	10 [28.6]	15 [42.8]	10 [28.6]	
Revised Geneva score (same for both reviewers)				0.001					0.914
Low	153 (19.4)	142 [92.8]	11 [7.2]		11 (10.1)	4 (36.4)	5 [45.5]	2 [18.1]	
Intermediate	574 (72.7)	493 [85.9]	81 [14.1]		81 (74.3)	23 (28.4)	33 [40.7]	25 [30.9]	
High	63 (8)	46 [73]	17 [27]		17 (15.6)	5 [29.4]	8 [47.1]	4 [23.5]	
Well's score: unlikely/unlikely	N=480				N=44				
D-dimer cut-off: 1000				0.013					0.037
<1000	123 (75.5)	115 [93.5]	8 [6.5]		8 (50)	5 [62.5]	2 [25]	1 [12.5]	
≥ 1000	40 (24.5)	32 [80]	8 [20]		8 (50)	0 [0]	5 [62.5]	3 [37.5]	
D-dimer cut-off: 500				0.037					0.263
< 500	92 (56.4)	87 [94.6]	5 [5.4]		5 (31.3)	3 [60]	2 (40)	0 (0)	
> 500	71 (43.6)	60 [84.5]	11 [15.5]		11 (68.8)	2 [18.1]	5 [45.5]	4 [36.4]	
D-dimer cut-off: age-adjusted				0.006					0.263
< age-adjusted	104(63.8)	99 [95.2]	5 [4.8]		5 (31.3)	3 [60]	2 [40]	0 [0]	
> age-adjusted	59 (36.2)	48 [81.4]	11 [18.6]		11 (68.8)	2 [18.2]	5 [45.5]	4 [36.3]	
YEARS algorithm: 0 items / 0 items	N=419				N=37				
D-dimer cut-off: 1000				0.024					0.016
<1000	106 (74.1)	99 [93.4]	7 [6.6]		7 (46.7)	5 [71.4]	1 [14.3]	1 [14.3]	
≥ 1000	37 (25.9)	29 [78.4]	8 [21.6]		8 (53.3)	0 [0]	5 [62.5]	3 [37.5]	

**D-dimer cut-off: 500**				0.028					0.165
**< 500**	78 (54.6)	74 [94.9]	4 [5.1]		4 (26.7)	3 [75]	1 [25]	0 [0]	
**> 500**	65 (45.5)	54 [83.1]	11 [16.9]		11 (73.3)	2 [18.2]	5 [45.5]	4 [36.3]	
**D-dimer cut-off: age-adjusted**				0.004					0.165
**< age-adjusted**	90(62.9)	86 [95.6]	4 [4.4]		4 (26.7)	3 [75]	1 [25]	0 [0]	
**> age-adjusted**	57 (45.5)	42 [79.2]	11 [20.8]		11 (73.3)	2 [18.1]	5 [45.5]	4 [36.4]	
**Revised Geneva score: low risk**	N=184				N=11				
**D-dimer cut-off: 1000**				0.592					0.100
**<1000**	48 (75)	45 [93.8]	3 [6.2]		3 (60)	3 [100]	0 [0]	0 [0]	
**≥ 1000**	16 (25)	14 [87.5]	2 [12.5]		2 (40)	0 [0]	2 [100]	0 [0]	
**D-dimer cut-off: 500**				1.000					0.100
**< 500**	41 (64.1)	38 [92.7]	3 [7.3]		3 (60)	3 [100]	0 [0]	0 [0]	
**> 500**	23 (35.9)	21 [91.3]	2 [8.7]		2(40)	0 [0]	2 [100]	0 [0]	
**D-dimer cut-off: age-adjusted**				1.000					0.100
**< age-adjusted**	42 (65.6)	39 [92.9]	3 [7.1]		3 (60)	3 [100]	0 [0]	0 [0]	
**> age-adjusted**	22 (34.4)	20 [90.9]	2 [9.1]		2 (40)	0 [0]	2 [100]	0 [0]	

Notes: (1) All variables are expressed in n (%) or [%]; (%) correspond to columns and [%] to rows; (2) D-dimer is in ng/mL; (3) Wells score <=4: PE unlikely; (4) Revised Geneva Score 0-3: low risk for PE; (5) For Well's criteria and YEARS algorithm, clinical probability for PE as independently assessed by reviewer 1 and reviewer 2 are presented as 'reviewer 1/reviewer 2'; (6) Age Adjusted D-dimer (Age x 10 microgram/Liter if age > 50 years)
Abbreviations: N: number, PE: pulmonary embolism

Supplementary File S4: D-dimer and PE

D-dimer and Pulmonary Embolism				
		**Pulmonary Embolism**		
**D-dimer**	**Total**	**No**	**Yes**	
	N=917	N=789	N=128	p-value
**D-dimer Absolute value MEAN(SD)**	1949.6 (5576.7)	1514.6 (5248.5)	4586.5 (6737)	<0.001
**D-dimer Absolute value MEDIAN(IQR)**	490 (277 -1298)	418 (260 -908)	1956 (607-5954)	<0.001
**D-dimer (ng/ml)**				<0.001
**<500**	175 (50.6)	166 [94.9]	9 [5.1]	
**500-1000**	70 (20.2)	62 (88.6)	8 (11.4)	
**>1000**	101 (29.2)	69 (68.3)	32 (31.7)	
**D-dimer (ng/ml)**				<0.001
**<1000**	244 (70.5)	228 (93.4)	16 (6.6)	
**>= 1000**	102 (29.5)	69 (67.6)	33 (32.4)	
**D-dimer (ng/ml)**				<0.001
**<500**	175 (50.6)	166 (94.9)	9 (5.1)	
**>=500**	171 (49.4)	131 (76.6)	40 (23.4)	
**D-dimer (Adjusted)**				<0.001
**Equal/Higher than D-dimer**	145 (41.9)	106 (73.1)	39 (26.9)	
**Lower than D-dimer**	201 (58.1)	191 (95.0)	10 (5.0)	

Notes: All variables are expressed in n (%)**.** Except for D-dimer Absolute value that is expressed in MEAN(SD) and D-dimer Absolute value that is expressed in MEDIAN(IQR).

Abbreviations: IQR: Interquartile range; N: number; PE: Pulmonary Embolism; SD: Standard Deviation; %: Percentage.

**Supplementary File S5: Patients with low clinical probability and D-dimer < 500**								
	**D-dimer**	**Age, Sex**	**Comorbidities**	**Type of PE**	**Presenting symptoms**	**Risk factors**	**Well's score**	**Geneva score**
**Well's score unlikely/unlikely + D-dimer < 500**								
**Patient #1**	243	46, Male	HTN, Asthma	Sub-segmental	Sudden-onset pleuritic chest pain x 2 days	Works as a driver (sits for 8-10 hours)	3	3
**Patient #2**	364	46, Female	Migraines	Sub-segmental	Sudden-onset pleuritic chest pain x 2 days	None	3	3
**Patient #3**	442	85, Female	HTN, DM, Stroke	Sub-segmental	Sudden-onset Afib and dyspnea	Hip fracture repair x 2 days ago	3	8
**Patient #4**	297	40, Female	None	Segmental	Sudden-onset dyspnea and pleuritic chest pain x 2 days with bilateral calf pain	Right ear surgery x 8 days	3	7
**Patient #5**	332	37, Male	HIV, Polysubstance abuse	Segmental	Syncope, palpitations, chest pain, dyspnea, leg pain	History of leiomyosarcoma complicated by blood clots in his heart	3	6
**YEARS algorithm: 0 items / 0 items + D-dimer < 500: Patients #1 to #4 same as above**								
**Revised Geneva score: low risk + D-dimer <500: Patients #1 and #2 same as above**								
**Patient #6**	470	41, Female	None	Sub-segmental	Pleuritic right-sided chest pain	In the hospital, undergoing treatment for complicated pyelonephritis with sepsis. Chest x-ray suggestive of COVID pneumonia (PCR negative)	n/a	3

Notes: (1) D-dimer is in ng/mL; (2) For Well's criteria, 'unlikely/unlikely' represents low clinical probability for PE as independently assessed by reviewer 1 and reviewer 2; (3) for YEARS algorithm, '0 items/0 items' represents low clinical probability for PE as independently assessed by reviewer 1 and reviewer 2; (4) revised Geneva Score 0-3: low risk for PE; and (5) D-dimer values are in ng/ml.

Abbreviations: Afib: atrial fibrillation; DM: diabetes mellitus; HTN: hypertension; N: number; n/a: not applicable due to missing values; and PE: pulmonary embolism
